# HELPING OR HINDERING: ASSOCIATIONS BETWEEN DAILY POSITIVE EXPERIENCES AND COGNITIVE PERFORMANCE

**DOI:** 10.1093/geroni/igae098.2108

**Published:** 2024-12-31

**Authors:** Dakota Witzel, Zachary Taylor, Eric Cerino, Robert Stawski, David Almeida

**Affiliations:** South Dakota State University, Brookings, South Dakota, United States; Northern Arizona University, Flagstaff, Arizona, United States; Northern Arizona University, Flagstaff, Arizona, United States; University of Essex, Colchester, England, United Kingdom; Pennsylvania State University, University Park, Pennsylvania, United States

## Abstract

Daily stressors have significant effects on cognitive health across the lifespan, but little is known about how daily positive experiences may facilitate better cognitive performance. Leveraging the third wave of a publicly available adult lifespan sample who completed a telephone assessment of cognitive function (i.e., executive function, episodic memory) and an eight-day daily diary protocol (n=1,172; 55.76%Women, Mage=62.71), we examined how the exposure, number of, and affective reactivity to daily positive experiences were related to executive function and episodic memory. Models adjusted for daily stressor exposure, age, education, marital status, and gender. Person means for exposure and number of positive experiences, as well as negative (NAR) and positive (PAR) affective reactivity (decreases or increases in negative or positive affect, respectively) were calculated across eight days. Linear regressions suggest that having a higher proportion of days with positive experiences, and a higher number of positive experiences were related to better executive function and episodic memory (ps<.05). Higher NAR to the exposure to and number of daily positive experiences was related to better executive function; only NAR to the exposure to daily positive experiences were related to better episodic memory (ps<.05). Higher PAR to the exposure to and number of daily positive experiences was related to poorer episodic memory only (ps<.05). Discussion will focus on the complex differential associations among affective reactions to positive experiences and cognitive function. Future directions should evaluate possible non-linear effects of reactivity and mechanisms underlying associations between daily positive experiences and cognitive health across the lifespan.

